# Influence of Graphite Layer on Electronic Properties of MgO/6H-SiC(0001) Interface

**DOI:** 10.3390/ma14154189

**Published:** 2021-07-27

**Authors:** Rafał Lewandków, Piotr Mazur, Artur Trembułowicz, Agata Sabik, Radosław Wasielewski, Miłosz Grodzicki

**Affiliations:** Institute of Experimental Physics, University of Wroclaw, pl. M. Borna 9, 50-204 Wrocław, Poland; Rafal.Lewandkow@uwr.edu.pl (R.L.); Piotr.Mazur@uwr.edu.pl (P.M.); Artur.Trembulowicz@uwr.edu.pl (A.T.); Agata.Sabik@uwr.edu.pl (A.S.); Radoslaw.Wasielewski@uwr.edu.pl (R.W.)

**Keywords:** MgO, 6H-SiC, semiconductor, valence band, XPS, UPS

## Abstract

This paper concerns research on magnesium oxide layers in terms of their potential use as a gate material for SiC MOSFET structures. The two basic systems of MgO/SiC(0001) and MgO/graphite/SiC(0001) were deeply investigated in situ under ultrahigh vacuum (UHV). In both cases, the MgO layers were obtained by a reactive evaporation method. Graphite layers terminating the SiC(0001) surface were formed by thermal annealing in UHV. The physicochemical properties of the deposited MgO layers and the systems formed with their participation were determined using X-ray and UV photoelectron spectroscopy (XPS, UPS). The results confirmed the formation of MgO compounds. Energy level diagrams were constructed for both systems. The valence band maximum of MgO layers was embedded deeper on the graphitized surface than on the SiC(0001).

## 1. Introduction

Silicon carbide (SiC) is one of the most suitable semiconductors for high-power and frequency electronic devices due to its unique physical properties. SiC has a wide band gap along with high values of electron mobility, thermal conductivity, and breakdown voltage. Further, SiC is distinguished by a low intrinsic carrier concentration. SiC, compared to conventional semiconductors, is also highly resistant to chemical and thermal degradation, allowing SiC-based electronic devices to operate under harsh conditions. Such devices include field-effect transistors (FETs) that are based on metal oxide semiconductor (MOS) structures. Therefore, the formation of a dielectric film on SiC is one of the steps in MOS structure creation. SiO_2_ appears to be a natural candidate for use in SiC-based structures due to its easy preparation. However, this oxide has a dielectric constant over three times lower than SiC. This means that the SiO_2_ dielectric gate for SiC-based transistors would be limited to high-field operation. It would result in a narrowing of the performance of such devices, the operation of which would be significantly below the breakdown field of SiC. Hence, oxides with a high dielectric constant are desirable for MOS structures, among which are Al_2_O_3_, Ga_2_O_3_, TiO_2_, HfO_2_, and MgO. The latter mentioned insulator has a dielectric constant similar to SiC and a large band gap [1,2]. Further, this oxide possesses a relatively high thermal conductivity and stability, which substantially benefits the operational security and stability of high-power devices [3]. Moreover, MgO has a little mismatch with the SiC, which is about 3% for the MgO(111) and SiC(0001) surfaces, allowing the preparation of a high-quality MgO-gate [4]. There are several ways to grow MgO layers, including molecular beam epitaxy, atomic layer deposition, or magnetron sputtering [4,5,6,7]. Many factors can affect the insulator/semiconductor interface properties, including substrate surface cleanliness, surface stoichiometry, number of defects in a substrate, quality of adlayers, and thin film deposition method. They can affect the conduction band offset (CBO), valence band offset (VBO) at the interface, and, consequently, the performance of MOS devices. A stoichiometry of SiC(0001) surface can be easily modified by thermal annealing under vacuum. This process can lead to a graphite layer formation that affects the electronic structure of the surface [8]. Graphite layers can be helpful in forming Ohmic contacts with SiC [9]. Herein, we report on a new approach to the MgO layers formation on 6H-SiC(0001) by a reactive evaporation method. We also show the impact of the interfacial graphite layer on the electronic properties of the interface using surface-sensitive techniques such as X-ray and UV photoelectron spectroscopies, which allow insight into the valence band and deeper energy levels.

## 2. Experimental Details

**Figure 1:**
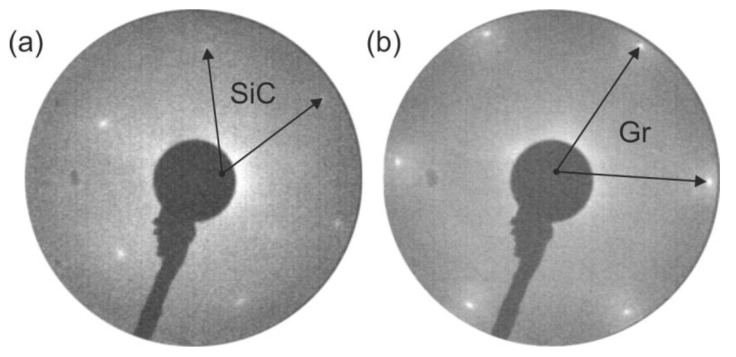
LEED patterns of (**a**) 6H-SiC(0001) (1 × 1) surface; (**b**) (1 × 1) graphite (Gr) obtained after annealing the surface from (**a**). Images taken for 79 eV. The arrows show the reciprocal unit vectors.

Silicon carbide samples (Cree, Durham, NC, USA), 10 × 5 mm^2^ in size, cut from a p-type 6H-SiC(0001) single crystal wafer terminated with 10-μm thick atomically flat homoepitaxial layers, were used in these experiments as substrates. Prior to transfer into the ultrahigh vacuum (UHV) chamber (SPECS, Berlin, Germany) with a base pressure lower than 1 × 10^−10^ Torr, the samples were chemically cleaned using a 10% HF solution and rinsed with distilled water. After being placed in UHV, they were degassed, then cleaned by rapid thermal annealing up to 600 °C. MgO films were formed in situ by the reactive evaporation method, magnesium (99.98% purity, from Sigma–Aldrich, Saint Louis, MO, USA) was evaporated from an electron beam evaporator with a flux monitor under an oxygen atmosphere (2.5 × 10^−6^ Torr), a similar method was previously applied for TiO_2_ films growth [10]. The thicknesses of the MgO layer was estimated from the dependence d = cosθ·λ·ln(I_0_/I), where θ is the angle between the normal to the substrate surface and the analyzer; I_0_ and I are the intensity values of the C 1s core-level line, respectively, before and after the deposition; and λ is the mean free path of the electron for MgO, similarly as in [11]. The interfacial graphite layer is based on the fact that during SiC annealing, the silicon atoms easily escape from the (0001) surface into the vacuum enriching it with carbon. This way allows for the creation of single layers of graphene as well as graphite [12,13,14,15]. The structure of initial surfaces before depositions was characterized by a low-energy electron diffraction (LEED) method. The obtained LEED patterns for 6H-SiC(0001) and (1 × 1) graphite together with their reciprocal unit vectors are presented in Figure 1. The graphite basis vectors are rotated by 30° to SiC, which is in agreement with previous studies [16,17]. The ratio between reciprocal lattice constants graphite/SiC is 1.2, and it is very close to the lattice mismatch between 6H-SiC(0001) (3.08 Å) and graphite (2.46 Å). The graphite thickness was 3 nm and was similarly estimated as for the MgO layers (based on the loss of the C–C component intensity in the C 1s line). This gives about five monolayers of graphene stacked on top of each other.

The MgO/SiC and MgO/graphite/SiC systems were characterized by an X-ray photoelectron spectroscopy (XPS) using a radiation source with an Mg K_α_ line (1253.6 eV) and ultraviolet photoelectron spectroscopy (UPS) with a He I line (21.2 eV) from a DC discharged lamp. Photoelectrons were collected using a hemispherical electron energy analyzer (Phoibos 100, Specs) with 0.1 eV and 0.025 eV energy steps and 10 eV and 2 eV pass energies for XPS and UPS techniques, respectively. The optical axis of the analyzer entrance was normal to the substrate surface. The position of the Fermi level (*E*_F_) was measured on an Ar-ion cleaned reference Au sample. Due to the charging of MgO layers during photoelectron experiments, binding energy (BE) calibration was performed according to the procedure proposed by Greczynski and Hutman in [18,19,20]. XPS and UPS spectra were analyzed using CasaXPS software. Deconvolutions of peaks were made using the Gaussian and Lorentzian line shapes and Shirley-type backgrounds. Measurements were taken at room temperature and made on freshly prepared layers to reduce exposure time to residual gases.

## 3. Results and Discussion

### 3.1. MgO/SiC(0001) System

**Figure 2:**
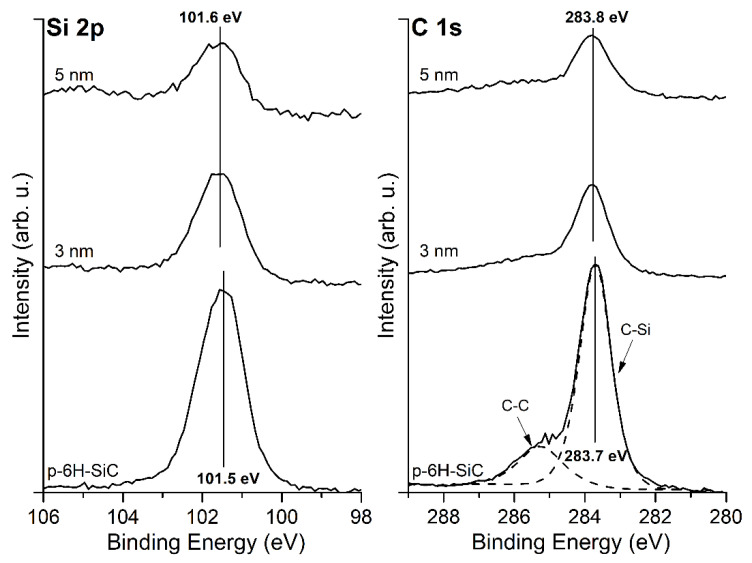
XPS spectra of Si 2p and C 1s core level lines for bare SiC(0001) surface and covered with MgO layers of an average thickness of 3 and 5 nm. Both peaks remain almost in the same positions after MgO deposition.

The shape of the Si 2p and C 1s core level lines for the bare SiC(0001) surface and its evolution with the MgO overlayer thickness is shown in Figure 2. The Si 2p for the cleaned surface is located at a BE of 101.5 eV. The line shifts by 0.1 eV towards a higher BE after the deposition of MgO layers. The C 1s peak contains two components, and the first is attributed to C–Si bonds, and the second to C–C bonds, which come from a carbon enrichment caused by thermal cleaning. During SiC annealing, Si atoms escape into the vacuum, which leads to the enrichment of the surface with carbon. The shape of the peak is similar to one previously obtained by us in other experiments [21]. The position of the C–Si component at a BE of 283.7 eV shifts slightly towards a higher BE by 0.1 eV after the deposition of the MgO peaks. Considering that the Si 2p and C 1s shifts are within the measurement accuracy, this proves that there is no significant electron transfer at the interface.

**Figure 3:**
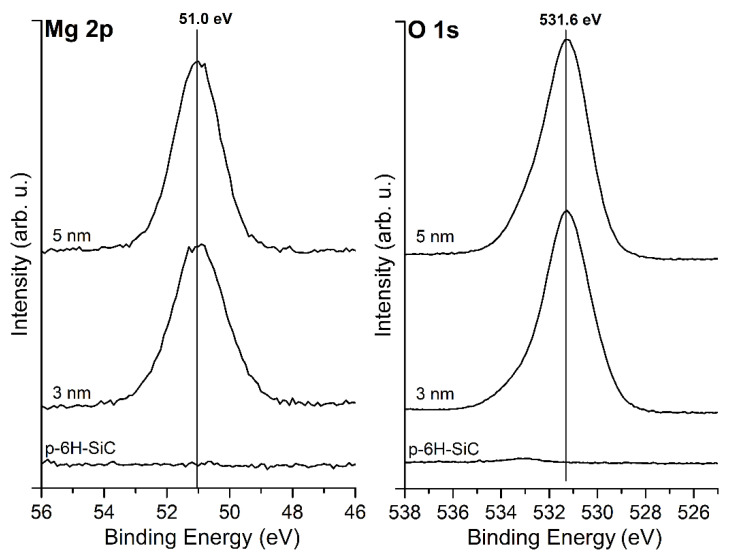
XPS spectra of Mg 2p and O 1s core level lines for bare SiC(0001) surface and covered with MgO layers of an average thickness of 3 and 5 nm. The peak positions correspond to the MgO compound and remain in the same binding energies regardless of the thickness.

**Figure 4:**
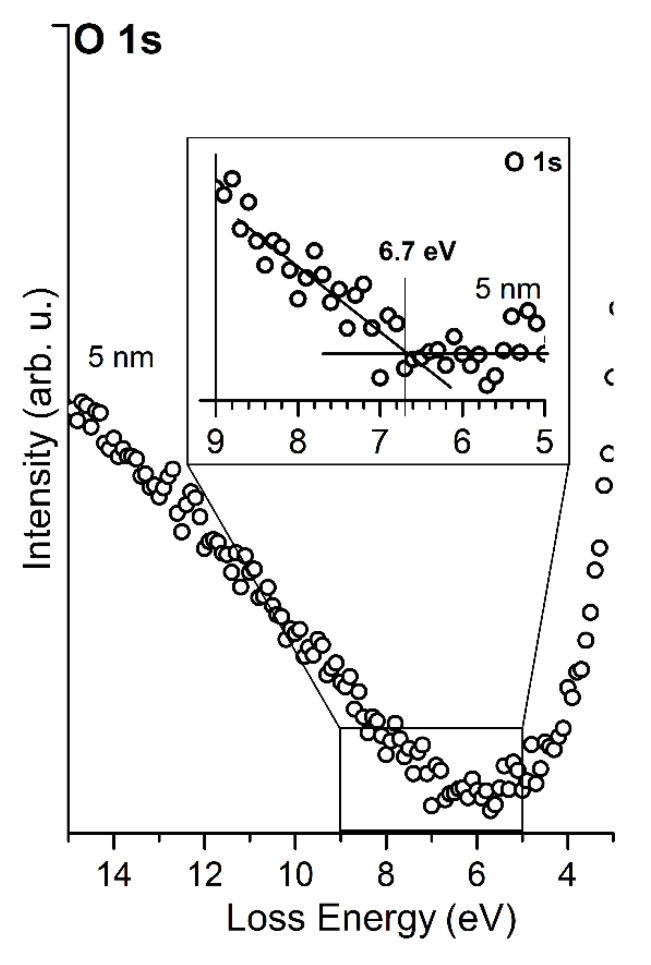
Band gap width determination of MgO layers from the onset of O 1s energy loss spectra. The average thickness of the layer is 5 nm.

The XPS analysis for MgO layers shows that the Mg 2p peak is located at a BE of 51.0 eV and O 1s has a BE of 531.6 eV, as shown in Figure 3. The Mg 2p and O 1s peaks remain in the same positions regardless of the thickness of MgO layers. The position of the Mg 2p peak corresponds to the Mg–O bonding and is shifted by about 1 eV towards a higher BE compared to magnesium in a metallic form. The location of the peaks is typical for a MgO compound [22,23,24,25]. This indicates that the deposition of MgO using a physical evaporation method under a UHV atmosphere enriched with oxygen is possible. An inelastic energy loss in the O 1s core-level line allows determining a band gap of the layers [26,27,28], as shown in Figure 4. In this case, the band gap was evaluated to be 6.7 eV for 5 nm thick MgO layers. The obtained values are typical for MgO in the form of thin layers and achieved by surface-sensitive techniques [29]. They are lower than the bulk band gap of MgO, which is assumed to be 7.8 eV. It is worth noting, however, that usually, the surface band gap is smaller than the bulk forbidden band [30], and in extreme cases, for example, very thin layers, may even be lower than 1 eV [29].

**Figure 5:**
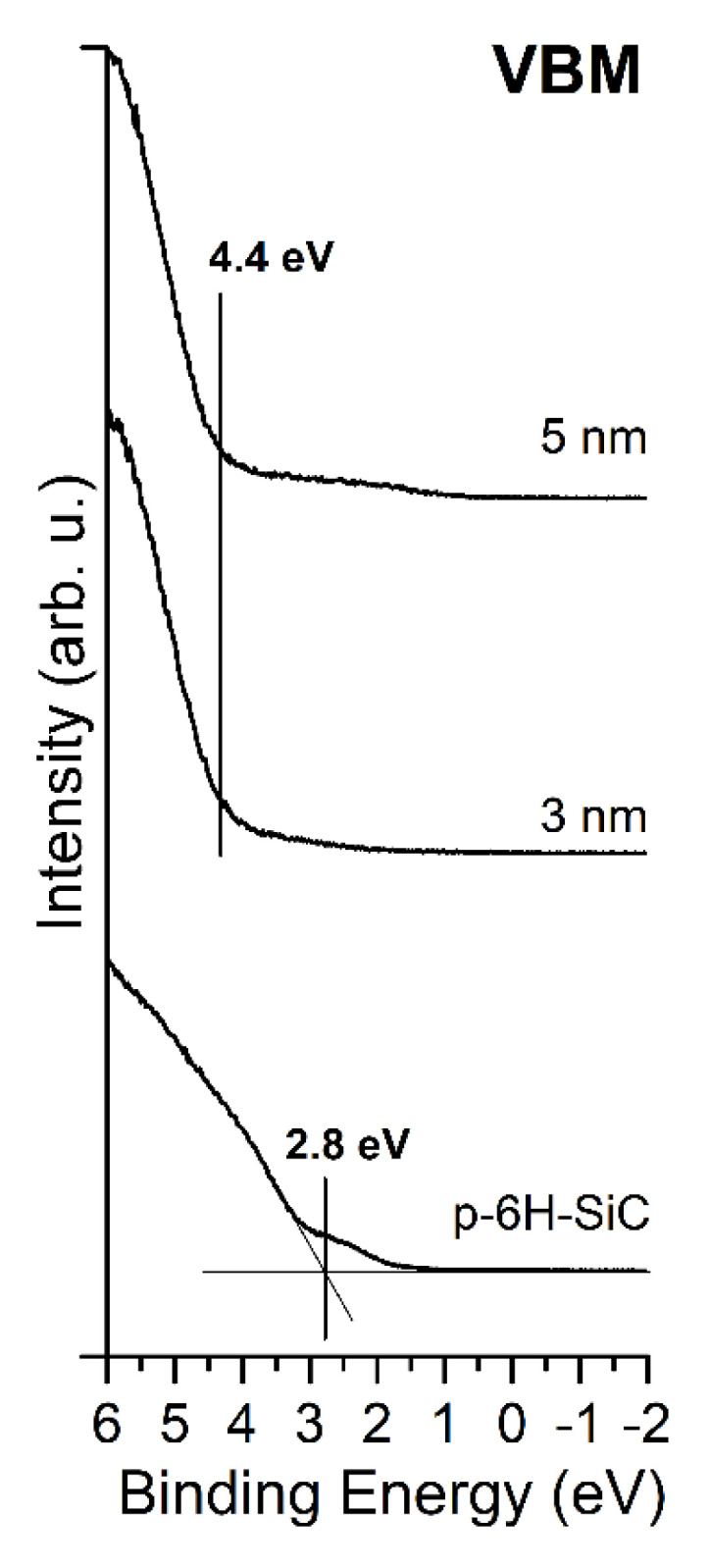
Evolution of UPS spectra for bare SiC(0001) surface and covered with MgO layers of an average thickness of 3 and 5 nm. The VBM of MgO remains in the same position for both coverages.

The valence band maximum (VBM) for the SiC substrate is located at 2.8 eV below the *E*_F_ and 280.9 eV above the C 1s core level. The latter value is in line with other works [31,32]. The deposition of MgO layers modified the electron energy distribution—the VBM is shifted to 4.4 eV, as shown in Figure 5.

### 3.2. MgO/Graphite/SiC(0001) System

**Figure 6:**
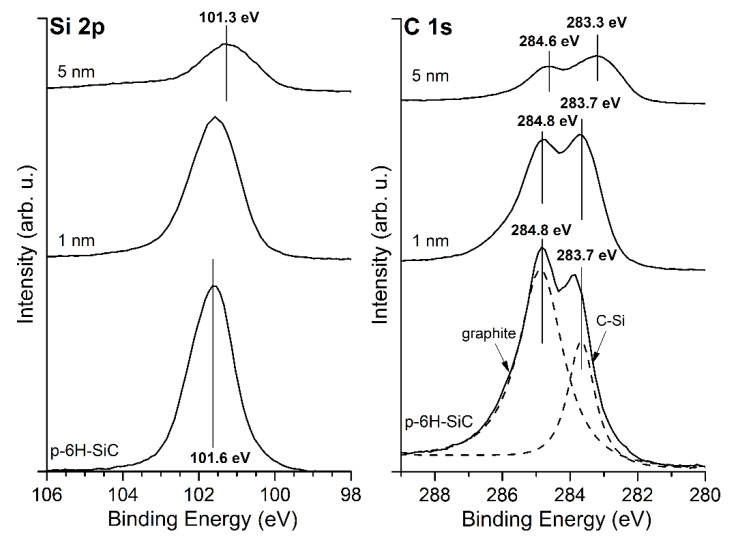
XPS spectra of Si 2p and C 1s core level lines for the bare graphite/SiC(0001) system and covered with MgO layers of an average thickness of 1 and 5 nm. For the bare graphite/SiC(0001), two components of C 1s peak, i.e., the C–C and C–Si bonding, are clearly visible.

The growth of MgO layers was repeated on the SiC(0001) substrate, which was earlier terminated with graphite layers obtained by thermal treatment under UHV. The Si 2p and C 1s core level lines for the graphite/SiC(0001) system and its evolution with a MgO overlayer thickness are shown in Figure 6. The Si 2p for the surface before MgO layers deposition is located at a BE of 101.6 eV. The line shifts towards a lower BE after the deposition of MgO layers, finally reaching the position of 101.3 eV.

The C 1s peak contains two components, first at a BE of 283.7 eV attributed to C–Si bonds, and second at a BE of 284.8 eV to C–C bonds, which come from the graphite layer. The position of the C 1s for the graphite phase is in line with [8,9]. The C 1s shifts towards a lower BE to the position of 283.3 eV and 284.6. After the deposition of MgO layers, the Mg 2p peak and O 1s are located at 51.0 eV and 531.6 eV, respectively. The positions are the same as for the layers deposited on the SiC(0001) sample presented in Figure 3. Another similarity is the width of the band gap; in the case of the graphite/SiC, the band gap of the MgO layers is 6.7 eV.

The value was also determined based on an inelastic energy loss in the O 1s core-level line, as was previously shown in Figure 4.

**Figure 7:**
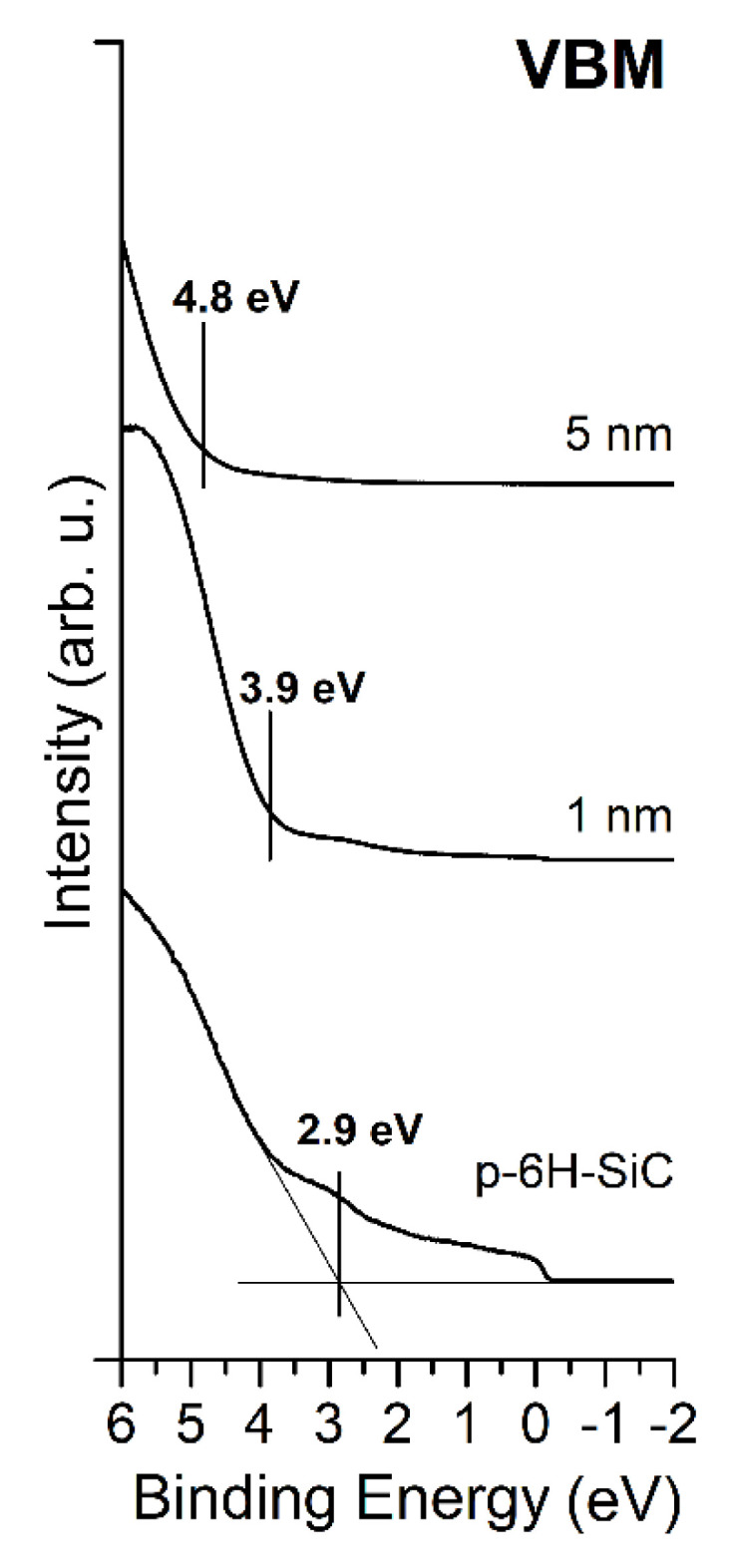
Evolution of UPS spectra for the bare graphite/SiC system and covered with MgO layers of an average thickness of 1 and 5 nm. In the vicinity of the *E*_F,_ the density of electron states from the graphite phase is clearly visible.

The UPS spectrum of the bare graphite/SiC system reveals the valence band edge from SiC as well as the Fermi tail from graphite layers, as shown in Figure 7. The VBM is located at 2.9 eV below the *E*_F_ and 280.8 eV above the C 1s core level. In the spectrum, the presence of graphite layers on SiC(0001) is manifested by the electron density of states beginning from the Fermi level. The deposition of MgO layers changed the electron energy distribution. The VBM is shifted to 4.8 eV.

## 4. Discussion

**Figure 8:**
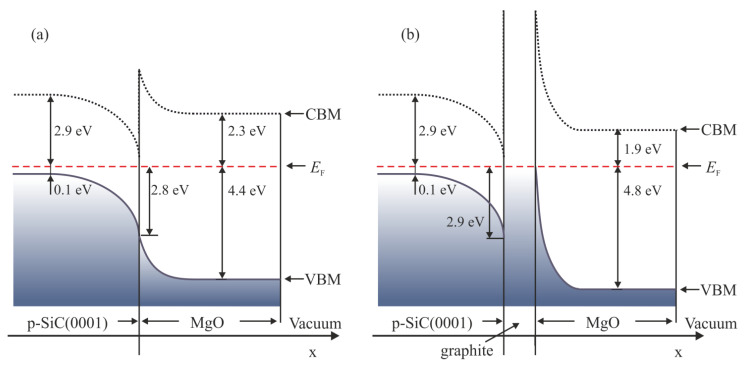
Illustrative energy level diagrams for (**a**) MgO/SiC and (**b**) MgO/graphite/SiC systems based on UPS measurements. In the sketches, the calculated depleted layer width is about 600 nm, and the highest thickness of MgO adlayers is 5 nm. The graphite interlayer is 3 nm thick.

The obtained data indicate that the growth of MgO layers by a reactive evaporation method is possible and allows for the comparison of the electronic structure of both systems. The UPS data enable construction sketches of electronic diagrams, as presented in Figure 8. The distance between the *E*_F_ and the valence band maximum in bulk is evaluated to be about 0.1 eV. In turn, the valence band maximum on the surface is located 2.8 eV below the *E*_F_. Thus for the bare SiC(0001) surface, a strong band bending (BB) is present. The magnitude of the BB is equal to 2.6 eV. The value is quite large and, taking into account that the investigated semiconductor is of p-type, it proves that the subsurface layers are heavily depleted. The width of the depletion region in the substrate was estimated to be about 600 nm. After the deposition of MgO layers, the VBM shifts to the final position of 4.4 eV below the *E*_F_. Given the obtained value of the band gap of 6.7 eV, the conduction band of the MgO layers is located 2.3 eV above the Fermi level and 0.6 eV below the conduction band of bulk SiC, as shown in Figure 8a. In the case of the graphitized SiC(0001) surface, i.e., graphite/SiC system, MgO layers have similar chemical properties. However, the UPS shows that the electron density of states in the vicinity of the Fermi level differs significantly due to graphite layers. The BB at the graphite/SiC interface has a similar magnitude. The Schottky barrier height for holes at the interface is 2.9 eV. After adding MgO layers, the VBM gradually shifted to 4.8 eV below the *E*_F_, as shown in Figure 8b. The valence band of the MgO layers is deeper, located by 0.4 eV lower than for the MgO/SiC system.

## 5. Conclusions

The results confirmed a new approach to the MgO layers formation by using a reactive evaporation method. XPS and UPS were used to investigate in situ the chemical and electronic properties of the MgO/SiC and MgO/graphite/SiC systems. For both systems, the chemical properties of MgO layers were the same. The Mg 2p and O 1s core level lines had positions of 51.0 eV and 531.6 eV. The positions of the core level lines for the substrate were typical. The C 1s was located at 383.7 eV for the C–Si bonding. At the initial surface, a strong band bending was observed. Its value was 2.7 eV. Comparing the data of the electronic structure diagrams for the two systems, we noted that the use of the interfacial layer of graphite led to a change in barrier shape between SiC substrate and MgO layers. The valence band of the MgO layers was located 0.4 eV deeper in the system with the graphite interlayer.

## Data Availability

The data presented in this study are available on request from the corresponding author.
